# Activation of apoptosis and G0/G1 cell cycle arrest along with inhibition of melanogenesis by humic acid and fulvic acid: *BAX/BCL*-2 and *Tyr* genes expression and evaluation of nanomechanical properties in A375 human melanoma cell line

**DOI:** 10.22038/IJBMS.2022.60651.13444

**Published:** 2022-04

**Authors:** Mitra Salehi, Hossein Piri, Alireza Farasat, Babak Pakbin, Nematollah Gheibi

**Affiliations:** 1 Student Research Committee, Qazvin University of Medical Sciences, Qazvin, Iran; 2 Department of Biochemistry and Genetics, School of Medicine, Qazvin University of Medical Sciences, Qazvin, Iran; 3 Cellular and Molecular Research Center, Research Institute for Prevention of Non-Communicable Diseases, Qazvin University of Medical Sciences, Qazvin, Iran; 4 Medical Microbiology Research Center, Qazvin University of Medical Sciences, Qazvin, Iran

**Keywords:** Apoptosis, BAX, BCL-2, Fulvic acid, Humic acid, Melanoma, Tyrosinase

## Abstract

**Objective(s):**

Humic acid (HA) and Fulvic acid (FA) are major members of humic substances, which are extracted from organic sources including soil and peat. The pro-apoptotic and anti-melanogenic effects of HA and FA at the cellular and molecular levels in the A375 human melanoma cell line were examined in this study.

**Materials and Methods:**

The cytotoxicity effect of HA and FA were evaluated by cell viability assay. Apoptosis and cell cycle were investigated by flow cytometry. Real-time PCR was carried out to measure the expression of *BAX*, *BCL-2,* and *Tyr* genes. Moreover, the changes in nanomechanical properties were determined through atomic force microscopy (AFM).

**Results:**

It was found that HA and FA decrease cell viability with an IC_50_ value of 50 µg/ml (dose-dependent) for 14 hr, arrested cells in the G0/G1 phase, and increased the sub-G1 phase (induce apoptosis). Based on the AFM analysis, Young’s modulus and adhesion force values were increased, also ultrastructural characteristics of cells were changed. Results of Real-time PCR revealed that HA and FA lead to a decrease in the expressions of *BCL-2* and *Tyr* genes, and increase the *BAX* gene expression.

**Conclusion:**

These results exhibited that HA and FA possess pro-apoptotic effects through increasing the *BAX/ BCL-2* expression in A375 cells. These molecular reports were confirmed by cellular nanomechanical assessments using AFM and flow cytometry. In addition, HA and FA inhibited melanogenesis by decreasing the expression of the *Tyr* gene. It is worthwhile to note that, HA and FA can be regarded to design new anti-cancer and anti-melanogenesis products.

## Introduction

Malignant melanoma is the most aggressive type of skin cancer (1). In 2021, the American Cancer Society appraised 106,110 new cases of this cancer in the USA. (2). The mortality rate of malignant melanoma accounted for more than 80% of all types of skin cancer; it has been revealed that current treatments of melanoma (localized radiotherapy, surgical resection, immunotherapy, and chemotherapy) are far from adequacy; this problem is related to metastasis to vital organs through blood vessels, resistance to current treatments, and poor early diagnosis (3). Therefore, it is necessary to prevent metastasis through early diagnosis and targeted therapies. In recent years, drug-resistance to targeted therapies for malignant melanoma has hindered researchers’ efforts (4).

One of the most important molecular markers in malignant melanoma is the tyrosinase enzyme; its structure contains carbohydrates (glycoprotein enzyme) (5). This enzyme restricted the oxidation of tyrosine in the melanogenesis pathway. The function of tyrosinase is regulated by a Microphthalmia-associated transcription factor or MITF that is an oncogene in the malignancy process of melanoma. Tyrosinase is downstream of MITF (6). To date, numerous studies have reported the anti-melanogenic effects of the natural compounds for inhibition of melanogenesis through decreasing the expression of *Tyr* and MITF genes. The previous study has revealed that targeting of the *Tyr* gene could be a strategy for the regulation of hyperpigmentation in melanoma cells (7, 8). 

Scientific evidence has confirmed that targeting intracellular signaling pathways such as RAS/BRAF/MAPK and PI3K/AKT in the treatment of malignant melanoma has not been adequate (3). Studies have suggested that targeting the *BCL-2* family is a proper strategy to control the malignancy of melanoma (9). Expression levels of *BCL-2*, *Bcl-xl*, *Bcl-w*, and *Mcl-1* (anti-apoptotic) and *BAX*, *Bak*, *Bad*, and *Bid* (pro-apoptotic) have a significant role in the regulation of the intrinsic apoptosis pathway. Studies have indicated that abnormal expression and dysregulation of the *BCL-2* family are the main reasons for resistance to the apoptosis pathway in metastatic melanoma (10). So far, inducing the apoptosis pathway through many compounds has been reported against metastatic melanoma cells *in vitro*. Depending on previous studies, numerous chemotherapeutics compounds originate from natural products (11). 

Currently, Atomic force microscopy (AFM) is used for assessment of the nanomechanical properties of cancer cells such as the morphological alteration of cells, cell-cell adhesion (prognosis of metastatic and benign cancer cells), and evaluation of apoptosis by formation of apoptotic bodies after treatment with anti-cancer agents (12). This microscopic assessment provides high image resolution (2D images) to evaluate changes in the ultra-structure of the cytoplasmic membrane in various cancer cells. Our study, investigated the nanomechanical properties of the A375 cell line after treatment with two organic acids by AFM (13).

Humic acid (HA), Fulvic acid (FA), and humin are major subclasses of organic substances known as humic substances or HSs. These compounds originate from the disintegration of organic material including dead plants in soil, peat, and aquatic environments through microbial metabolism (14). The structure of HSs has been explicated according to the analysis of Nuclear magnetic resonance (NMR) and Electron paramagnetic resonance (ESR) spectroscopy (15). HA is the main member of HSs; what segregates this organic acid from FA is a set of different physicochemical properties such as solubility, molecular weight (MW), and structure (14). The structure of HA mainly consists of aromatic rings, phenolic, carboxylic, alkoxyl, and quinone groups. HA is not soluble in strong acids but entirely soluble in alkaline media (16). Researchers have reported various effects of HA in health-promotion such as anti-inflammatory, ultraviolet protective properties, anti-oxidant, anti-neoplastic, and pro-apoptotic effects in several cancer cell lines (17). FA is the second member of HSs that has a smaller MW compared with HA and is soluble under all pH conditions (14). Results from previous studies confirm the presence of aromatic rings, phenolic, ketone, alkoxyl, and quinone groups in the FA structure (18). It has various pharmacological and biological activities; positive effects on chronic inflammatory diseases including diabetes, antibacterial activity, and anti-oxidant effect. Recent information has shown that FA effectively inhibits the cell viability of human cancer cell lines such as MCA-102 fibrosarcoma cells, Hep3B, LNCaP, and HL60 cells (14, 19).

According to the previous studies, HA and FA are effective for regulating the apoptosis pathway in cancer cells such as cervical cancer cells (20), Hep G2 cells (21), and HL-60 cells (22). But so far, the pro-apoptotic and anti-melanogenic effects of these compounds on human melanoma cells have not been discussed in the other studies. In our study, the pro-apoptotic and anti-melanogenic effects of HA and FA in the A375 human melanoma cell line through cellular and molecular assessments were examined. In molecular assessment, expression of *BAX*, *BCL-2*, and *Tyr* genes was carried out by Quantitative reverse transcription-polymerase chain reactions (qRT-PCR). In the cellular assessment, the triggering of apoptosis and cell cycle arrest in the presence of HA and FA was accomplished by flow cytometry. Furthermore, the change in nanomechanical properties of these cells is depicted by AFM. 

## Materials and Methods

***Chemicals and reagents***

HA (Cat. No. 53680) and Methylthiazolyldiphenyl-tetrazolium bromide (MTT) (Cat. No. M5655) were taken from Sigma-Aldrich Co (St. Louis, MO, US). FA was taken from Santa Cruz Biotechnology Co (Cat. No. sc-202615). Total RNA Mini Kit from Favor Prep company (Cat.NO. FABRK 001), cDNA Synthesis Kit (Cat. No. YT4500) and YTA SYBR Green qRT- PCR Master Mix 2X (Cat No. YT2551) were obtained from Yektatajhiz Co (Tehran. Iran). The Annexin V-FITC Apoptosis Kit (Cat. No. BMS500FI-100) was taken from Thermo Fisher Scientific. Dulbecco’s modified Eagle’s medium (DMEM/HEPES, F-12) (Cat. No. 11330032), FBS (Cat. No. 11573397), and Gibco™ antibiotics (Cat. No. 15070063) were taken from Thermo Fisher Scientific.

***Preparation of HA and FA***

Firstly, HA was purified through the base–acid method based on the study of Tsai *et al*. HA was dissolved in 1N NaOH solution (pH > 10), and the filtered solution was acidified until a pH of 1.5. For precipitate of HA, it was centrifuged (3000 ×g for 30 min) and then dissolved in 1N NaOH solution; later than the third round of base-acid treatments (alkaline–acid treatment), the HA was dissolved in NaOH (0.1N) and pH of the solution was reached to 7.2–7.4. Finally, the HA was freeze-dried to a powder (23). Then, lyophilized forms of HA and FA were dissolved in phosphate-buffered saline (PBS) (pH 7.4) to make a stock solution (100 µg/ml).

***Cell culture***

The human melanoma cell line (A375) (ATCC® CRL-1619) was obtained from the Pasteur Institute (Tehran, Iran). Cells were cultured in DMEM supplemented with 10% FBS and Gibco™ antibiotics (5000 U/ml of Penicillin /Streptomycin). Throughout all experiments, melanoma cells were maintained in supplemented DMEM medium and incubated at 37 °C in a humidified incubator with 5% CO_2_.

***Cell viability analysis by MTT assay***

To appraise the cytotoxicity of HA and FA on the A375 cell line, the cultured cells (5× 10^3^ cells/well) were incubated after seeding into the 96-well plates for 24 hr. After overnight incubation, the previous DMEM medium was pulled out, and to wash the cells, PBS (pH 7.4) was added to the wells. Then cells were treated with HA and FA (0, 5,10,20,50, and 100 µg/ml) at 14 hr in the humidified incubator. Next, after pulling out the previous medium and washing with PBS, 100 µl of MTT solution (0.5 mg/ml) was added to the wells and incubated for 3-4 hr at 37°C in the humidified incubator (dark cell culture room). After observing the deposits on the wells, to dissolve the formazan precipitate, 100 µl of dimethyl sulfoxide (DMSO) was added to the wells. Finally, the absorbance was read through microplate readers (Biotech Instruments, USA). To calculate the IC_50 _values of HA and FA, Graph Pad Prism program was used (24).

***Detection of cell apoptosis***

In our study, to assess the pro-apoptotic effects of HA and FA in the A375 cell line, cultured cells (5 × 10^5^ cells/well) were seeded into the ﬂat-bottomed 6-well plates and incubated 24 hr to the cells adherent to the bottomed of well plates. After overnight incubation, the previous DMEM medium was pulled out and the cells were washed with PBS. Subsequently, cells were treated with concentrations (50 and 100 µg/ml) (the most effective concentrations to decrease the cell viability that were measured by the MTT assay) of HA and FA for 14 hr at 37 °C. After pulling out the previous medium, the other steps were performed based on the instructions of the kit. In short, all cells (floated and adherent cells) were centrifuged at 1500 rpm for 5 min. After observing the cell pellet, they were washed with cold PBS and 300 µl of binding buffer (1x) was added. After adding 2 μl of FITC-Annexin, the samples were incubated for 15 min without light; and the samples were read after adding 1 μl of PI. The samples were detected by BD FACS Calibur (BD Biosciences, San Jose, CA, USA) (25). 

***Cell cycle analysis***

To evaluate the effect of HA and FA on the cell cycle, 5 × 10^5^ cells were seeded into the wells of ﬂat-bottomed 6-well plates and incubated for 24 hr, then the previous medium was pulled out and the cells were washed with PBS, and the cells were treated with concentrations 50 and 100 µg/ml of HA and FA for 14 hr at 37 °C. Afterward, at first all cells (adherent and floating) were centrifuged (1500 rpm for 5 min), then the cell pellet was washed with PBS (two times) and 250 µl of prepared master mix solution (20 μg/ml of RNase A and 50 μg/ml of PI) was added and incubated at 37 °C for 1–2 hr. Finally, DNA content was detected by a BD FACS Calibur (BD Biosciences, San Jose, CA, USA) through PI fluorescence detected in the FL3 channel (red channel). The results were analyzed through Multicycle software (Partec PAS, Münster, Germany) (25).

***Atomic force microscopy (AFM)***

At first, to prepare the samples for AFM analysis, cultured cells of the A375 cell line (2 × 10^5 ^cells) were seeded into the ﬂat-bottomed 60 mm Petri dishes and were incubated for 24 hr. After overnight incubation, adherent cells were treated with concentrations 50 and 100 µg/ml of HA and FA at 14 hr. Subsequently, cells were washed with PBS and fixed by glutaraldehyde solution (0.5 %) for 1 min; then glutaraldehyde solution was pulled out and cells were washed with PBS three times (5min for each time), in the end, PBS was removed and the cells dried at room temperature (24).

After preparation of the cells, to reveal the effects of HA and FA on nanomechanical and morphological properties of cells, AFM was performed using a Hitachi AFM 5100N with a V-shaped and High Sensitivity Tip (nominal spring constant 0.35-0.07 N/m, side angle 10 ^○^, and 10 nm radius) (MicroMasch-NSC15/AIBS) attached to the cantilever. Data analysis was performed using Scanning Probe Image Processor (SPIP) (6.7.9) software. At first, cells were placed at room temperature (6–12 hr), and in the parallel, the AFM device was calibrated in non-contact mode at 25 ^○^C and 40% of humidity; images were taken from different areas of the sample at least 10–35 separate and measurable cells from each of selected specified ranges. Finally, cellular adhesion force and Young’s modulus were calculated based on pulling measurements and force-displacement curves (cantilever vertical deflection or interaction forces between the probe and cell) and also the following formula (24): 

$F=\frac{2}{\pi }\tan(a)\frac{E}{1-v^{2}}\delta^{2}$

 (26)

=Poisson ratio 0.5 is appropriate for cells, F= Loading force, = Indentation, E= Young’s modulus, = Half opening angle of a conical tip.

***RNA extraction and qRT-PCR method***

The qRT-PCR was performed to find the efficacy of HA and FA on the mRNA levels of *BAX*, *BCL-2*, and *Tyr *genes. Based on the protocols of the total RNA extracted kit, 10^6^ cells/well were seeded into the ﬂat-bottomed 6-well plates and were incubated for 24 hr in order to adhere the cells to the bottom of the plates. After overnight incubation, the medium was pulled out and after washing the cells with PBS, the cultured cells were treated with concentrations 50 and 100 µg/ml of HA and FA for 14 hr at 37°C. Subsequently, Moloney Murine Leukemia Virus Reverse Transcriptase (M-MLV RT) was used for synthesis of complementary DNA (cDNA) from extracted total RNA based on the protocols of the kit. Quantity and quality of extracted total RNA and cDNA were examined through NanoDrop spectroscopy (Epoch Microplate Spectrophotometer, Bio Tek instruments; USA) and agarose gel electrophoresis (1.5%). The qRT- PCR reactions were carried out according to YTA SYBR Green qRT- PCR Master Mix 2X kit on Rotor-Gene Q 2plex HRM Platform (Cat No./ID: 9001560). For quantification of gene (*BAX*, *BCL-2*, and *Tyr*) expressions, the 2^-ΔΔCT^ method (fold changes of genes expression) was carried out. The cyclin conditions and the sequences of the primers are shown in (Tables 1 and 2) (25). 

***Statistical analysis***

Data was statically analyzed using one-way ANOVA. In our study, statistical analysis of results was carried out via Statistical Package for the Social Sciences software (SPSS) (version 20; SPSS Inc, Chicago, IL, USA) and GraphPad Prism software (version 9.1.0.221); the result was considered statically significant difference at *P*<0.05* and *P*<0.01**.

## Results

***HA and FA decreased cell viability***

**Figure 1:**
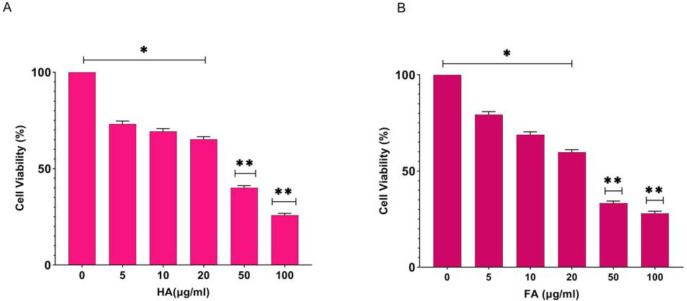
Effects of Humic acid (HA) and Fulvic acid (FA) on cell viability in A375 human melanoma cell line. (A) Effect of HA on cell viability by MTT assay; (B) Effect of FA on cell viability by MTT assay. Data were statically analyzed through one-way ANOVA (*P*<0.05*; significant difference and *P*<0.01**; highly significant difference)

The cytotoxicity of HA and FA on the A375 cell line was investigated through MTT assay as explained in the Materials and Methods. The results are shown in (Figures 1A and B). According to these results, HA and FA decreased cell viability (dose-dependent) with an IC_50_ value of 50 µg/ml, and also the cell viability in 100 µg/ml concentration was significantly decreased. Next, 50 and 100 µg/ml concentrations of HA and FA at 14 hr were selected for other sections of the study.

***HA and FA induced the apoptosis pathway***

**Figure 2:**
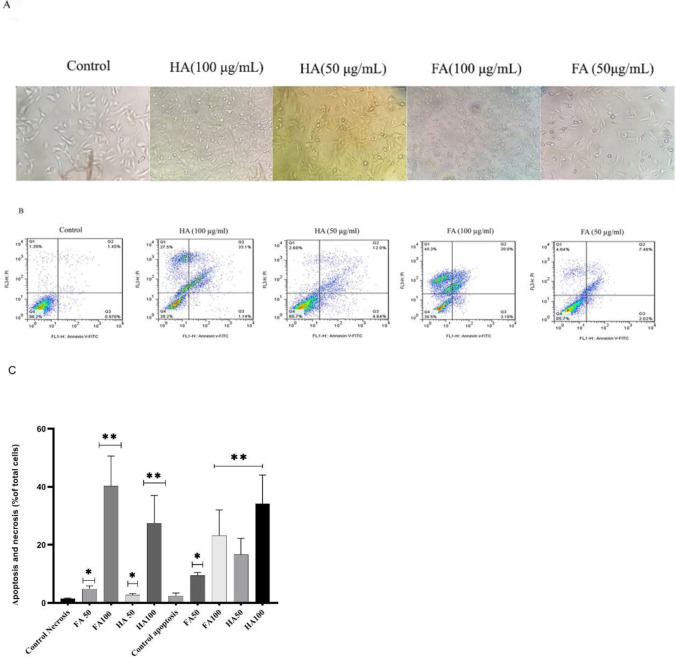
Pro-apoptotic effect of Humic acid (HA) and Fulvic acid (FA) in the A375 human melanoma cell line with the flow cytometry method. (A) Cultured cells after incubation (14 hr) with HA and FA under an inverted microscope; along with increasing the Concentrations of HA and FA, the rate of total apoptotic cells, necrotic cells, and also number of apoptotic body formations were increased. (B) and (C) Results of flow cytometry after incubation of cells (14 hr) with HA and FA. Data was statically analyzed through ANOVA (*P*<0.05*; significant difference and *P*<0.01**; highly significant difference)

The pro-apoptotic effect of HA and FA (50 and 100 µg/ml) for 14 hr on cultured cells was investigated through V-FITC/Pi Apoptosis Detection Kit that is explained in the Materials and Methods. As shown in (Figures 2A, B, and C), after treatment of cells with HA and FA, apoptosis is increased compared with untreated cells (control). Figure 2A depicts apoptotic body formation increase after treating cells with HA (100 µg/ml) and FA (100 µg/ml) for 14 hr. Also, along with an increase in the Concentrations of HA and FA, the rate of total apoptotic cells especially late apoptotic cells and necrosis cells significantly increased (Figures 2B and C). 

***HA and FA induced the G1 arrest***

**Figure 3:**
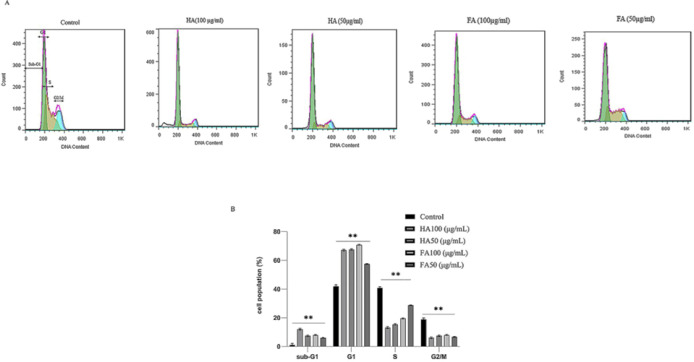
Cell cycle analysis of A375 human melanoma cell line after treatment with Humic acid (HA) and Fulvic acid (FA) (50 and 100 µg/ml) for 14 hr. (A) and (B) Analysis the results of DNA content with the flow cytometry method demonstrated that both HA and FA arrests treated cells in G0/G1 phase compared with the control group; and also increased peak of sub-G1 phase or DNA fragmentation in treated cells confirmed that both of them could trigger the apoptosis pathway. Data were statically analyzed through ANOVA (*P*<0.05*; significant difference and *P*<0.01**; highly significant difference)

Cell cycle regulation by HA and FA was determined by analyzing the results of DNA content with flow cytometry. According to Figures 3A and B, after incubation of cells with HA and FA (50 and 100 µg/ml), it was found that both HA and FA (two concentrations) arrested cells in G0/G1 phase compared with untreated cells (control group). Simultaneously, after treatments of cultured cells, the accumulation of cells in the S phase substantially decreased. Our results confirmed that treatments of cells with HA and FA induced apoptosis via increased peak of the sub-G1 phase (Figures 3A and B).

***Nanomechanical properties***

**Figure 4:**
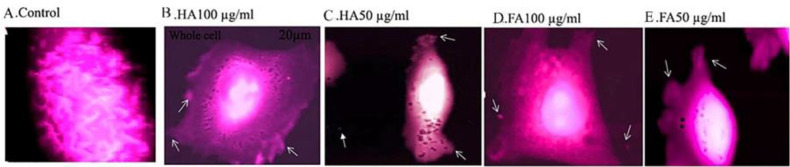
Whole-cell 2D AFM images of cellular change (ultrastructure of the cytoplasmic membrane of A375 cell line) after being treated with Humic acid (HA) and Fulvic acid (FA) for 14 hr. (A) control; (B) HA 100 µg/ml; (C) HA 50 µg/ml; (D) FA 100 µg/ml; and (E) FA 50 µg/ml. White arrows on figures depict apoptotic bodies

The efficacy of HA and FA for altering the nanomechanical and morphological properties of the A375 cell line to reduce metastasis of these cells was analyzed by AFM *in vitro*. According to Figure 4, treating cells with HA and FA (50 and 100 µg/ml) significantly led to changes in the ultrastructure of the cytoplasmic membrane of the A375 cell line compared with the control group; also 2D images revealed that the number of apoptotic bodies increases along with the increase in the concentration of HA and FA (dose-dependent effect). According to the values of Young’s modulus and cell-cell adhesion force, treated cells with HA and FA (100 µg/ml) have more cell-cell adhesion force compared with the 50 µg/ml concentration and the control group (Table 3).

***Gene expression***

**Figure 5:**
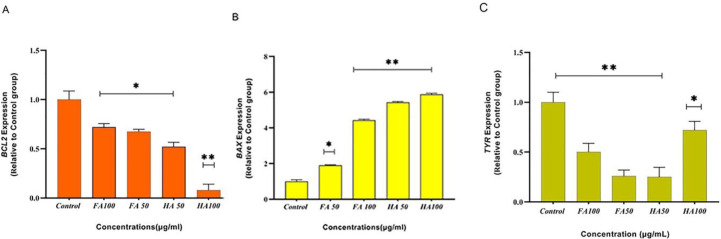
Evaluation of pro-apoptotic and anti-melanogenic effects of Humic acid (HA) and Fulvic acid (FA) in the A375 human melanoma cell line with the qRT-PCR method. (A) Decreased expression of the *Bcl-2* gene after treatment with HA and FA; (B) Increased expression of the *Bax* gene after treatment with HA and FA; (C) Decreased expression of the *Tyr* gene after treatment with HA and FA. Data was statically analyzed through ANOVA (*P*<0.05*; significant difference and *P*<0.01**; highly significant difference)

The qRT-PCR technique was done to evaluate the molecular mechanism of the pro-apoptotic and anti-melanogenic effects of HA and FA (50 and 100 µg/ml) on the A375 cell line. Analysis of results through the 2^-ΔΔCT^ method (fold changes of genes expression) demonstrated that, after treating cells with HA and FA (50 and 100 µg/ml) for 14 hr, the expression of *BCL-2* and *Tyr* genes substantially decreased while the expression of the BAX gene increased (dose-dependent effect). Therefore, HA and FA at the 100 µg/ml concentration have more pro-apoptotic and anti-melanogenic effects on the A375 cell line compared with the 50 µg/ml concentration (Figures 5A, B, and C). 

**Table 1.** Sequences of the primer pairs for qRT-PCR. (F, forward; R, reverse)

Size (bp)	Primer	Gene
**102**	F:5′- GCCTCCTCTCCTACTTTG-3′ R:5′- CTCAGCCCATCTTCTTCC-3′	***Bax***
**298**	F:5′- TGGGAAGTTTCAAATCAGC-3′ R:5′- GCATTCTTGGACGAGGG-3′	***Bcl-2***
**135**	F:5′-CAATGACCCCTTCATTGACC-3′ R:5′-TGGAAGATGGTGATGGGATT-3′	***GAPDH***
**130**	F:5′-TGCCAACGATCCTATCTTCTT-3′ R:5′-TTCCCGGTTATGTCCAATGG -3′	***Tyr***

**Table 2:** Cyclin conditions for qRT-PCR

Time	Temperature	Cycle	Stage
**5 min**	95 °C	1cycle	**Initial denaturation**
**40 sec**	95 °C	30 cycle	**Stage1(Denature)**
**40 sec**	55 °C		**Stage2 (Anneal)**
**40 sec**	72 °C		**Stage3 (Extend)**

**Table 3:** Changes in nanomechanical parameters of human melanoma cell line (A375)

Compounds	Mean Young’s modulus value(kpa) ± SE	Mean Adh^*^. Force (pN) ± SE	Mean Z^**^ pulling (µm) ± SE	*P*-Value
Control	3.2±0.09	203±12.2	1.56±0.06	-
FA50	6.6±1.89	225.3±14.8	2.06±0.06	0.051
FA100	8.32± 2.3	242±13.9	1.23±0.04	0.032
HA50	7.52±1.9	253±11.2	1.01±0.03	0.012
HA100	12.32±3.2	278±16.3	2.2±0.02	0.022

*Adh. Force = adhesion force, ** Z pulling =pulling distance

## Discussion

Based on the results of our study, HA and FA have anti-proliferation, pro-apoptotic, and anti-melanogenic effects on the A375 cell line; also, these agents change the cellular nanomechanical properties of the A375 cells. With respect to the pro-apoptotic effect of HA in cervical cancer cells (20), Hep G2 cells (21), and HL-60 cells (22), this study was designed to evaluate the anti-cancer effects of HA in the A375 cell line. In the current study, the results of the MTT assay demonstrated that HA and FA decrease the cell viability (IC_50_ value: 50 µg/ml), also significantly decreased cell viability at 100 µg/ml for 14 hr (Figures 1A and B). To evaluate the pro-apoptotic effects of HA and FA in the A375 cell line at the cellular level, an annexin V- FITC/PI test and cell cycle analysis were conducted. The results demonstrated that both of them significantly induce the apoptosis pathway in a dose-dependent manner on cultured cells of the A375 cell line (Figures 2A, B, and C). The cell cycle analysis based on (Figures 3A and B) revealed that both HA and FA arrested treated cells in the G0/G1 phase and therefore blocked the cell cycle progression from G1 to S-phase; these results offer that HA and FA inhibit cell proliferation through blocking the cell cycle; also increased peak of sub-G1 phase or DNA fragmentation in treated cells confirmed that both HA and FA trigger the apoptosis pathway compared with the control group. The effect of FA-CM (culture medium of FA-stimulated RAW 264.7 cells) on cell viability, apoptosis, and cell cycle progression in the MCA-102 cell line through MTT assay, DNA fragmentation, and flow cytometry are reported by Jayasooriya *et al*. They exhibited that increasing the DNA fragmentation and the sub-G1 phase after treatment of MCA-102 cells with FA-CM confirmed the pro-apoptotic effects of FA in these cells (19). Similar to our results, Yang *et al*. reported that incubation of RAW264.7 cells with HA (100 and 200 µg/ml) increased the peak of the sub-G1 phase and as a result triggered the apoptosis pathway and arrest of treated cells in the G2/M phase; they for clear these results was measured Bax, Bcl-2, caspase-9, and caspase-3 proteins by western blotting. Overall, the results of their study revealed that incubation of RAW264.7 cells with HA triggers the apoptosis pathway (27). Also, Lee *et al*. demonstrated that incubation of fibroblast C3H10T1/2 cells with HA arrest treated cells in the G0/G1 phase, and as a result, inhibits cell proliferation by blocking the cell cycle in G0/G1 phase (28).

Metastasis is a dynamic process controlled by different adhesion molecules and cytoskeletal dynamics in cancer cells. Previous studies have shown that the values of Young’s modulus and cell-cell adhesion force were being reduced in metastatic cancer cells. In our study, the results of AFM demonstrated that HA and FA increased the values of Young’s modulus of elasticity and cell-cell adhesion force in the A375 cell line (Table 3). Dutta *et al*. reported that benign cells of breast cancer (MCF-10A) compared with malignant breast cancer (MCF-7 and MDA-231) have higher Young’s modulus values in normoglycemic conditions (29). Moreover, Mei *et al*. demonstrated that treatment of B16-F10 melanoma cells with cisplatin alters morphology, actin cytoskeletal dynamics, and cytoplasmic membrane ultrastructure of cells. Also, the results of their research demonstrated that with an increase in the concentration of cisplatin, Young’s modulus was decreased; therefore it was found that B16-F10 melanoma cells are resistant to cisplatin (30). Based on Figure 4, treatment of the A375 cell line with HA and FA induces morphological alterations in the ultra-structure of the cytoplasmic membrane; also leading to an increase in the numbers of apoptotic bodies of cells compared with the control group. These results confirmed the pro-apoptotic effect of HA and FA in the A375 cell line.

Subsequently, the qRT-PCR technique was performed to evaluate the molecular mechanisms by which HA and FA affect the apoptosis pathway. Apoptosis is mostly regulated by *BCL-2* proteins. Another experiment has demonstrated that *BCL-2 *(powerful apoptosis inhibitor) and *BAX* (heterodimerizes and inhibits *BCL-2*) have a crucial effect on triggering the apoptosis pathway (31, 32). Previous reports demonstrated that treatments of HL-60 cells and human umbilical vein endothelial cells (HUVECs) with HA induce the apoptosis pathway via down-regulation of *BCL-2* and up-regulation of *BAX* genes (22, 27). In our study, analysis results of qRT-PCR revealed that incubation of the A375 cell line with HA and FA in both concentrations (50 and 100 µg/ml) leads to induction of apoptosis via a decrease in the mRNA levels of *the BCL-2* gene (anti-apoptosis) and increase the mRNA levels of the *BAX* gene (pro-apoptosis) (Figures 5A and B). Based on another finding, the pro-apoptotic effects of HA and FA are probably related to the change of oxidative stress in cells through disruption of the outer membrane of mitochondria (33). Previous studies confirmed that HA triggers the apoptosis pathway in the human promyelocytic leukemia HL-60 cells and human cervical cancer cells via increasing the levels of Reactive Oxygen species (ROS) (ROS-Mediated Mitochondrial Pathway) (20, 22). Also according to Schepetkin *et al*., FA leads to production of ROS in peritoneal macrophage cells (34). 

Tyrosinase is a regulatory enzyme in melanin biosynthesis (melanogenesis) and is expressed in the melanocyte and melanoma cells. Studies have demonstrated that in the metastatic melanoma cells the expression of tyrosinase is increased and down-regulation of the* Tyr* gene can affect inhibition of cell proliferation, promote apoptosis, and reduce hyperpigmentation by affecting signaling pathways such as the RAS/BRAF/MAPK (35-37). According to the study conducted by Chen *et al*., Hispolon (a polyphenolic compound) by decreasing the protein levels of Tyrosinase and MITF inhibited the melanogenesis in B16F10 cells, also this compound via increasing protein levels of caspase 3, 8, and 9 triggered apoptosis (38). Our results demonstrated that incubation of the A375 cell line with HA and FA decreased expression of the *Tyr* gene, and as a result inhibited melanogenesis, also triggering apoptosis (Figures 5A, B, and C). Therefore, further studies are needed to explain the possible association between decreasing expression of the *Tyr* gene and stimulation of cellular apoptosis in the presence of HA and FA in the A375 cell line. 

## Conclusion

It was found that HA and FA have potential pro-apoptotic effects in the A375 cell line through increased expression of* BAX *and decreased expression of* BCL-2* genes; these molecular reports were confirmed by results of flow cytometry and AFM. Results of AFM demonstrated that in the presence of HA and FA the cell-cell adhesion force and numbers of apoptotic bodies in the A375 cell line are increased*.* Assessments of anti-melanogenic effects of HA and FA were carried out by measuring expression of the *Tyr* gene; results have shown that expression of the *Tyr* gene is decreased after treatment by HA and FA. Based on these results, further experiments are needed for the measurement of the ROS level after treatment with HA and FA in the A375 cell line (ROS-mediated mitochondrial pathway). Overall, our study suggested that HA and FA could be useful for the design of new anti-cancer and anti-melanogenesis products. 

## Authors’ Contributions

NG and HP Designed the study; MS, BP, and AF Performed laboratory tests, data collection, analysis of results, drafting, and final checking of the manuscript. The authors declare that all data were generated in-house and that no paper mill was used. 

## Conflicts of Interest

The authors of this study have no conﬂicts of interests.
